# Down Syndrome and Alzheimer's Disease in a Dish: A multidimensional front‐door spanning pathogenesis and therapeutics

**DOI:** 10.1002/alz70855_107425

**Published:** 2025-12-25

**Authors:** Julie R. Korenberg

**Affiliations:** ^1^ University of Utah, Salt Lake City, UT, USA

## Abstract

**Background:**

Down syndrome (DS) is a leading model for studying therapeutic implications of co‐occurring conditions, including Alzheimer's disease (AD), intellectual disabilities (ID), and disturbances in cardiovascular (CV), pulmonary (P), and immune (Im) systems. These conditions cause significant suffering and remain unmet medical needs. Recent advances in iPSCs (induced pluripotent stem cells) derived from individuals with DS have helped accelerate research translation to clinical care.

**Method:**

This study presents a unique panel of iPSCs designed to explore the contributions of distinct chromosomes, genomic genes, and gender to AD, ID, and CV, P, and Im systems. The Utah iPSC collection includes 17 cell lines from individuals with DS, most of which have paired fibroblast and lymphoblastoid cell lines. These individuals underwent deep phenotyping, including multimodal neural imaging, cognition assessments, and data from seven independent Pan‐Omics databases. The panel includes samples from identical twins discordant for DS, partial trisomies (three without APP duplication), and individuals with genetic variants such as apolipoprotein e2/e2. We used proteomics, phosphoproteomics, RNA sequencing, microRNA analysis, metabolomics, and lipidomics to examine gene influences, including APP, IFNAR, ITSN1, DYRK1A, TTC3, DSCAM, MMU17, and their homologs.

**Result:**

The iPSC panel provides valuable insights into AD, DS, and related systems by distinguishing gene influences. Integration of 14 multidimensional datasets—spanning behavioral, cognitive, and neural imaging data—offers a detailed view of brain function, behavior, and organ development. This comprehensive approach allows for the dissection of AD and DS pathogenesis and the modeling of multiple human organs and cell types

**Conclusion:**

This work creates a comprehensive resource to understand DS at the organismal, cellular, and systems levels. The Utah iPSC collection is an invaluable tool for accelerating research translation into therapeutic strategies to improve the quality of life for individuals with DS and related conditions.

